# Ultrastructural and histopathologic findings after pars plana vitrectomy with a new hypersonic vitrector system. Qualitative preliminary assessment

**DOI:** 10.1371/journal.pone.0173883

**Published:** 2017-04-11

**Authors:** Salvador Pastor-Idoate, Richard Bonshek, Luciane Irion, Isaac Zambrano, Paul Carlin, Aleksandr Mironov, Paul Bishop, David McLeod, Paulo Eduardo Stanga

**Affiliations:** 1 Manchester Royal Eye Hospital, Central Manchester University Hospitals NHS Foundation Trust, Manchester Academic Health Science Centre, Manchester, United Kingdom; 2 Manchester Vision Regeneration (MVR) Lab at Manchester Royal Eye Hospital and NIHR/ Wellcome Trust Manchester CRF, Manchester, United Kingdom; 3 National Specialist Ophthalmic Pathology Service, Manchester Royal Eye Hospital, Manchester, United Kingdom; 4 Eye Bank, Manchester Royal Eye Hospital, Manchester, United Kingdom; 5 Operating Theatre Services, Manchester Royal Eye Hospital, Manchester, United Kingdom; 6 Electron Microscopy Core Facility, Faculty of Life Sciences, University of Manchester, Manchester, United Kingdom; 7 Faculty of Biology, Medicine and Health, University of Manchester, Manchester, United Kingdom; Massachusetts Eye & Ear Infirmary, Harvard Medical School, UNITED STATES

## Abstract

**Purpose:**

Preliminary assessment of a new prototype ultrasound-based hypersonic vitrector (HV) by qualitatively examining the histopathological changes in the retina and vitreous body after pars plana vitrectomy (PPV) and its ability to fragment vitreous collagen.

**Methods:**

Fourteen porcine cadaveric eyes, 20 eyes in live swine and six human cadaveric eyes underwent PPV using the HV or a pneumatic guillotine vitrector (GV). An additional 4 porcine crystalline lenses were touched with either the HV or GV for 1 minute. Following PPV, human vitreous was removed and processed for electron microscopy (EM). Eyes and lenses were fixed and sectioned for light microscopy (LM).

**Results:**

There were no macroscopic retinal or optic nerve defects associated with either HV or GV PPVs. Cadaveric retinal specimens showed separation of the inner limiting membrane (ILM) and vacuolization and fragmentation at the nerve fiber layer (NFL) and the ganglion cell layer (GCL). ILM fragmentation and separation were found after PPV in live swine with both vitrectors. Small disruptions of the posterior capsule or structural lens defects were found after HV touch. The EM analysis revealed more fragmentation of human vitreous collagen fibrils after HV compared to GV PPV.

**Conclusions:**

LM and EM analysis of retina, vitreous, and crystalline lens after PPV showed similar morphological changes using the HV or the GV. Vitreous fragmentation appeared more effective with the HV. Overall this study suggests that the HV may be a promising new technology. More work is needed to quantitatively assess its safety and efficacy.

## Introduction

The goal of all vitreous surgery is to minimize collateral damage while maximizing efficiency [1]. Advances in technique and instrumentation have supported a significant reduction in incision size, allowing faster healing and decreasing the probability of hypotony. A concomitant disadvantage is a decrease in flow rate and sometimes greater vitreous traction with smaller gauge vitreous cutters [1]. Traditionally, the focus for improvement in cutting vitreous has been on altering cut speed, fluidics, duty cycle, cutting port surface, or the diameter of the internal shaft in pneumatic, guillotine vitrectors (GV) [2–5]. However, there may be an upper limit to the achievable speed of the vitreous cutter blade [6].

A promising alternative to GV is the application of ultrasound (US) to liquefy and excise the vitreous [6–8]. US technology is widely accepted for vitrectomy during complicated cataract surgery to remove lens fragments from the posterior cavity with an US fragmentation handpiece [9–12]. Low power US harmonics may provide an alternative to GVs.

The hypersonic vitrectomy system uses low amplitude ultrasonic (US) motion of the tip to create oscillating high speed flows near the port that ‘cut’ vitreous. It also liquefies the vitreous in the vicinity of the tip to the viscosity of water. This allows the hypersonic vitrector (HV) to address some of the limitations of GVs. The US HV has a single needle instead of two needles, so there is no chance of trapping vitreous strands between the port edge and the needle. The port is continuously open, allowing smaller port sizes and larger inner-lumen diameters. This, in turn, lowers flow resistance and infusion pressures.

One concern with the use of US energy in vitreoretinal surgery is the extent of the effect of US on the vitreous and the potential for retinal lesions in the vicinity of the US vitrector. The mechanism of US damage to the retina is unknown. Acoustic waves like US radiation are mechanical waves, but respond to optical laws. Transmission or reflection and absorption are the major phenomena taking place at tissue interfaces [13]. Correspondingly, energy absorption may be transformed into heat. Apart from that, high energy US in liquids leads to "cavitation", a process producing microexplosions that may cause mechanical destruction [14]. US probes at the retina can produce retinal lesions [15]. It is possible that the apical retinal pigmented epithelial (RPE) cell layer represents an acoustic tissue interface at which reflection and absorption of ultrasonic waves takes place [15–17].

The purpose of this study was to assess the morphological and histological changes to the retina, vitreous and crystalline lens produced after pars plana vitrectomy (PPV) with an US-based hypersonic vitrector (HV) compared to a GV.

## Methods

All human tissue samples were treated in accordance with applicable laws for research involving human tissues and samples (MRC 2004-Biomedical Research; Manchester Eye Bank, Human Tissue Authority Licence 11056) and in accordance with the Declaration of Helsinki. The study using animal and human cadaveric tissues and samples was approved by the Manchester Royal Eye Hospital Steering Committee (R03781, Nov 2014). The porcine experiments were approved by the Research and Ethics Committee of the Animal Welfare and Ethical Review Body and the Home Office inspector (PPL-50/2506) of the University of Manchester (Manchester, United Kingdom), were conducted according to the principles of the Animal Scientific Procedures 1986 Act, Ec86/609 Directive 2010/63/EU, and adhered to the Association for Research in Vision and Ophthalmology statement for the use of animals in ophthalmic and vision Research.

### Vitrectors

**Fig 1:**
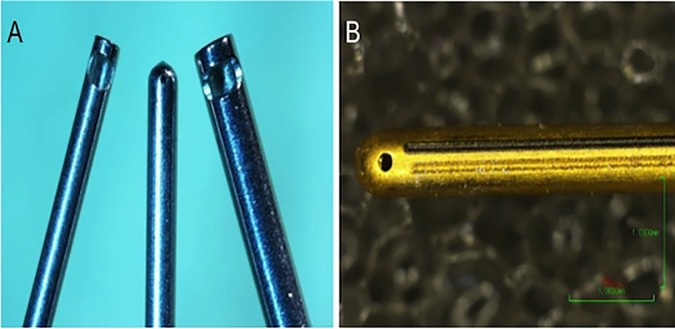
Comparison of the HV vs. conventional guillotine vitreous cutter. (A) 23-gauge hypersonic vitrector needle (center) compared with 25-gauge (right) and 23-gauge (left) of guillotine needle. (B) High-magnification image of the tip and port of the hypersonic vitrector.

The GV used in the study was a commercially available Bausch & Lomb, 23 gauge, pneumatically driven vitrector, operated off of the Bausch & Lomb Stellaris PC Vision Enhancement System (Fig 1A). The HV was a prototype electrically driven vitrector with a piezoelectric transducer element yielding an operating frequency of 28.5 kHz (Fig 1A and 1B). The energy range use to drive the HV in this study is less than 5% of the US energy used in traditional lens fragmentation. The HV was also operated off of the Bausch & Lomb Stellaris PC Vision Enhancement System, using prototype HV control hardware and software.

### Test parameters

We considered that every increment of 1000 CPM in the GV corresponded to an increment of 10% of US power in the HV, being the minimum set at 1000 CPM/10%US and the maximum set at 5000 CPM/50% US power.

### Porcine cadaveric eye

**Fig 2:**
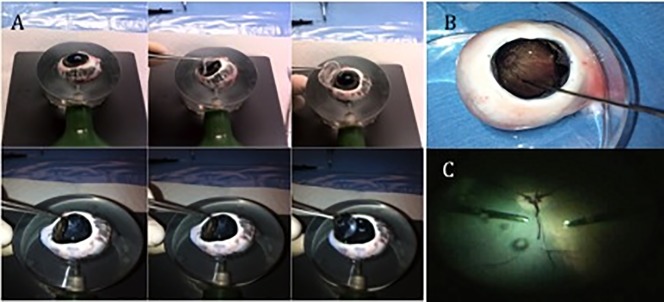
Open-sky and closed Pars Plana Vitrectomy (PPV) procedures in porcine cadaver eyes. (A) Removal of the cornea, crystalline lens and iris “en bloc” from the eye. (B) Porcine cadaver eye ready for the open-sky vitrectomy, showing the core vitreous and the posterior pole without any retinal detachment. (C) Closed PPV with the hypersonic vitrector through the 23-gauge trocar system.

Fourteen cadaveric porcine eyes were obtained within 12–24 h of local slaughter (Nixon’s Farm Shop Cheadle SK8 3PS, Manchester, UK). All eyes were kept in balanced salt solution (BSS) at 4°C until used. Eyes underwent either closed or open-sky PPVs. To perform the open-sky vitrectomy any residual periorbital tissue was surgically removed and the sclera trephined 4 mm from the limbus using a 15° Laseredge Stab Knife (B +L Storz Ophthalmic, St. Louis, MO, USA), forceps and surgical scissors. The cornea, iris and crystalline lens were removed “en bloc” (Fig 2A). The eyes were positioned in a holder so that the trephined area was located superiorly, allowing performance of an open-sky vitrectomy (Fig 2B). Closed PPVs were performed using a 23 G trocar system (Stellaris PC Vitrectomy system, Bausch + Lomb, St. Louis, MO, USA) (Fig 2C).

In all PPVs, the vitrector (GV or HV) was held 3 to 5 mm in front of the macula and in front of the optic nerve head (ONH). Both systems used 23 G vitrectors and Venturi vacuum pumps for aspiration at levels of 50 to 600 mmHg.

**Fig 3:**
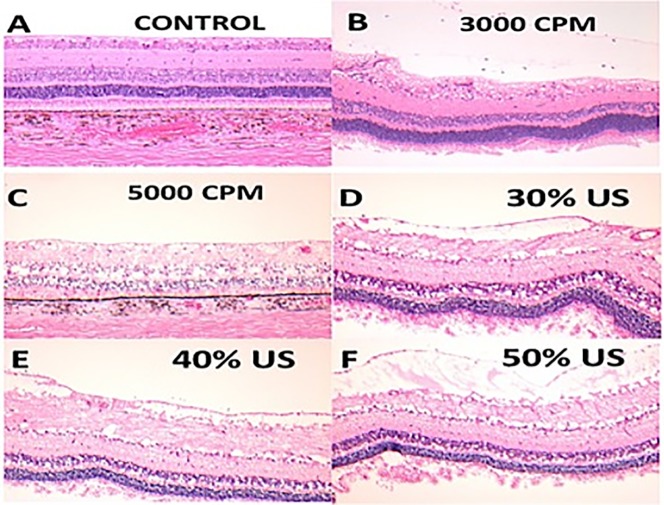
Hematoxylin-eosin stained horizontal retinal sections from porcine cadaveric eyes after pars plana vitrectomy procedures. (A) Section from a control eye without any procedure. (B and C) Retinal sections from eyes that underwent pars plana vitrectomy with a guillotine vitrector at 3000 or 5000 cuts per minute. ILM separation (B) and minimal disintegration at inner retinal layers (B, C). (D, E, and F) Retinal sections from eyes that underwent pars plana vitrectomy with a hypersonic vitrector at settings of 30% (D), 40% (E), and 50% (F) US power showing vacuolization, fragmentation of the nerve fiber and ganglion cell layers and inner limiting membrane separation without any disruption. (A, B D and E) Sections from a open-sky vitrectomy procedures. (C and F) Sections from a closed vitrectomy procedure.

**Fig 4:**
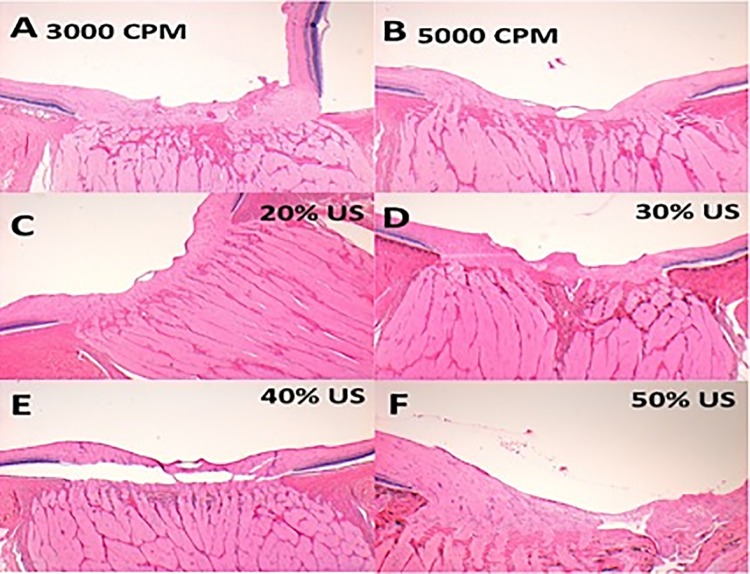
Hematoxylin-eosin stained sections through the optic nerve head from porcine cadaveric eyes after PPV. (A and B), Sections from eyes that underwent pars plana vitrectomy using the guillotine cutter at 3000 cuts per minute (A) or 5000 cuts per minute (B). (C, D, E and F), Sections from eyes that underwent pars plana vitrectomy using the hypersonic vitrector set at 20% (C), 30% (D), 40% (E), and 50% (F) ultrasound power. (A) and (F) show a subhyaloid haemorrhage and retinal detachment. E shows presumed artifactual detachment. (A, C, D and E) Sections from a open-sky vitrectomy procedures. (B and F) Sections from a closed vitrectomy procedures.

Six eyes underwent closed PPV: 5 eyes with the HV set to 10%, 20%, 30%, 40%, or 50% power and 1 eye with GV set at 3000–5000 CPM as a control. Seven eyes underwent open-sky PPV: 5 eyes with the HV set to 10%, 20%, 30%, 40%, or 50% power and 2 eyes with the GV, 1 at 3000–5000 CPM and 1 at 1000–3000 CPM. One final eye underwent the open-sky procedure without a PPV as a further control (Figs 3A–3F and 4A–4F).

### Porcine cadaveric crystalline lens

**Fig 5:**
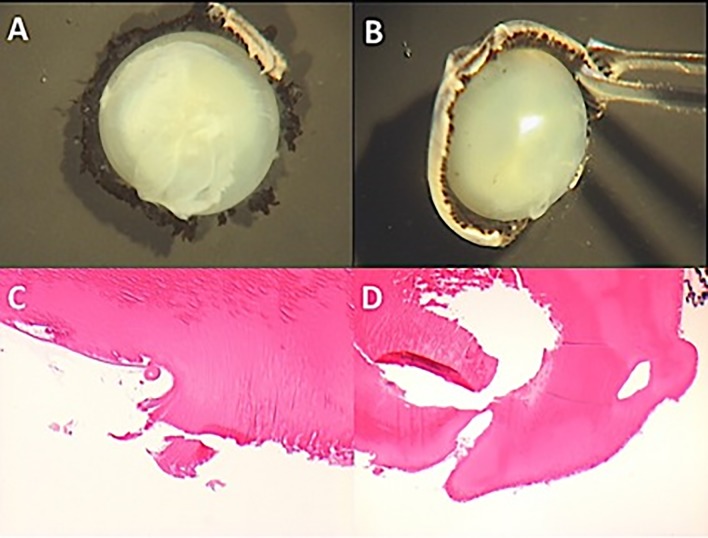
Macroscopic figures showing the effects of touching a guillotine. (A) or hypersonic (B) vitrector to the posterior capsule of a cadaveric porcine crystalline lens. Microscopic analysis showing disruption of the posterior capsule of the lenses is extensive with the guillotine vitrector (C) and focal with the hypersonic vitrector (D). Artifactual loss of tissue is also seen in (D).

Four cadaveric porcine crystalline lenses were positioned in holders and submerged in BSS. The crystalline lenses at the posterior capsule were touched for one minute each with the HV and GV (Fig 5A–5D).

### Human cadaveric eyes

**Fig 6:**
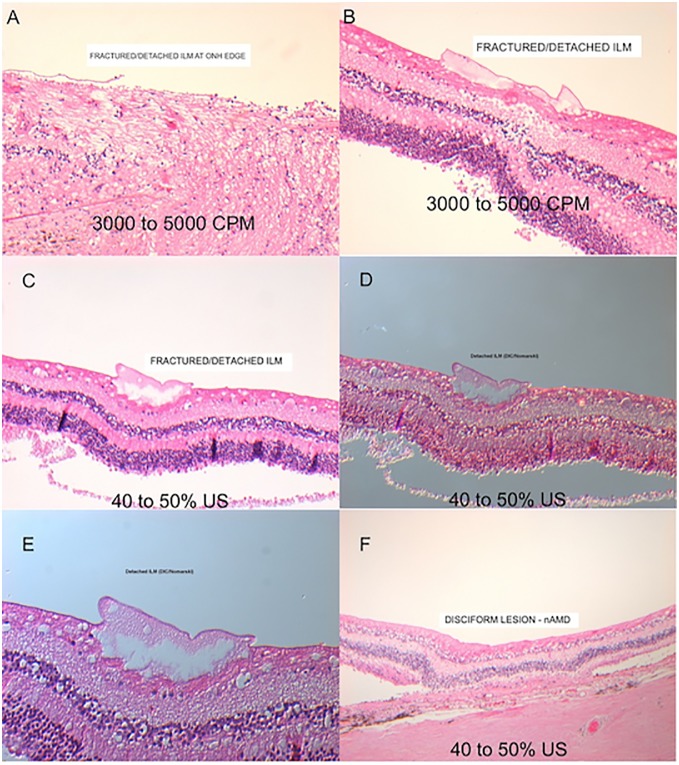
Hematoxylin-eosin stained histological sections of human retina after pars plana vitrectomy. All sections (A-C and F) show fragmentation and separation of the inner limiting membrane. (A and B) vitrectomy with a guillotine vitrector at 3000–5000 cuts per minute. (C) vitrectomy with a hypersonic vitrector at 40%–50% ultrasound power. (D-E) Differential interference contrast microscopy to enhance the contrast of ILM. (E) magnified image showing ILM fragmentation. (F) vitrectomy with a hypersonic vitrector at 40%–50% ultrasound power. Disciform macular degeneration.

Six post-mortem human eyes (Eye Bank at Manchester Royal Eye Hospital, Manchester, UK) from subjects 30–80 years of age with no reported history of retinal surgery were obtained. Open-sky, 23 G vitrectomy was performed with the HV in four eyes and the GV in two eyes. Explanted eyes were immersed in ice-cold medium and transported on ice to the laboratory where, under aseptic conditions, each eyeball was bisected with scissors, dividing the ocular globe into anterior and posterior eye-cups. The posterior eye-cups were positioned in a holder that allowed performance of an open-sky vitrectomy as in porcine eyes. The HV and GV were held 3–5 mm in front of the macula. Aspiration levels were 300–600 mmHg for both systems. Cut rates for the GV were 3000–5000 CPM and US power for the HV was set to 40% or 50% (Fig 6A–6F).

Differential interference contrast (DIC) microscopy technique was used to enhance the visualization and the contrast of the defects in ILM (Fig 6D–6E).

**Fig 7:**
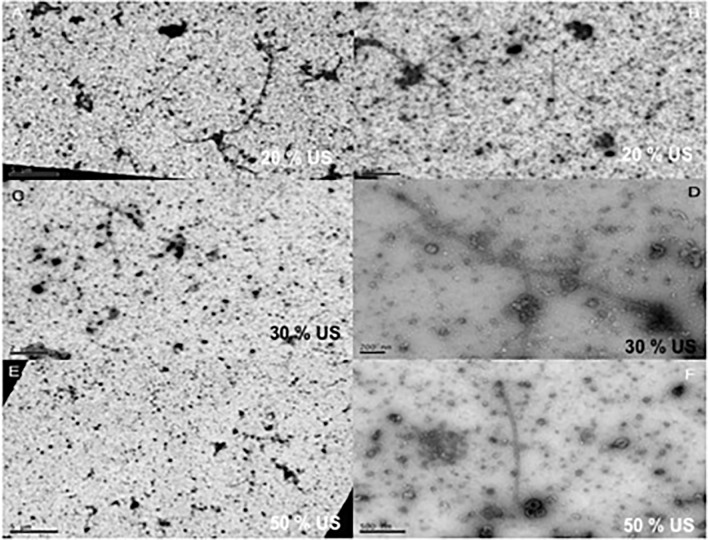
Transmission electron microscopy of human vitreous samples after pars plana vitrectomy with the hypersonic vitrector. (A and B) Vitrectomy at 20% ultrasound power showing fragmentation of the human vitreous collagen fibrils. The levels of collagen fibril fragmentation increased with increasing ultrasound power. (C and D) at 30% ultrasound power. (E and F) at 50% ultrasound power.

**Fig 8:**
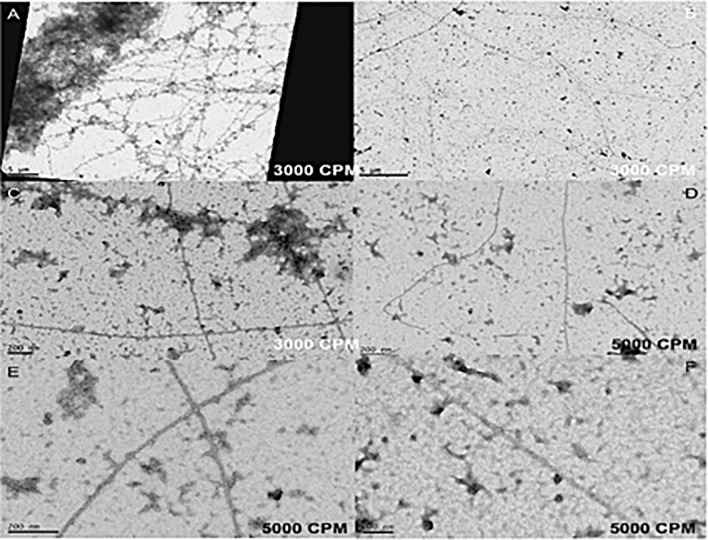
Transmission electron microscopy of human vitreous samples after pars plana vitrectomy with the guillotine vitrector. (A, B, C) GV at 3000 cuts per minute. (D, E, F) GV at 5000 cuts per minute. The figures show less fragmentation of the collagen fibrils compared to those obtained from the hypersonic vitrector shown in Fig 7.

In addition, in the early stages of each vitrectomy surgery, undiluted chopped vitreous samples were collected using a vitreous trap technique [18] for EM and negative staining analysis of the effects of the HV and GV on the collagen fibrils of chopped vitreous (Figs 7A–7F and 8A–8F).

### Live porcine vitrectomy

Twenty eyes from ten 20-week-old Landrace commercial swine (Centre for Integrative Mammalian Biology, Faculty of Life Sciences, University of Manchester, Manchester, UK) weighing 15–20 kg were used in the study. The swine were anesthetized with a mixture of 4% isoflurane on 100% O_2_ via face mask, intubated and maintained on isoflurane to effect and 100% O_2_. The concentration of isoflurane was the lowest required to produce full surgical anaesthesia, between 2.5% and 3%. Levels of anaesthesia and animal welfare were monitored via capnography and pulse oximetry.

The vitrectomy was performed through three 23 G ports (Stellaris PC Vision Enhancement System, Bausch + Lomb, St. Louis, MO, USA) by insertion of trocars on the sclera 4 mm from the limbus, 3 clock-hours apart. One port was used for the infusion cannula connected to a sterile bottle of BSS, the second port for the vitrector and the third one for the hand illumination.

A widefield fundus non-contact lens was placed with clear gel on the cornea to aid in visualizing the peripheral retina. A complete core vitrectomy was performed and the vitreous close to the retinal surface at the macula was removed, holding the vitrector (HV or GV) 3–5 mm in front of the macula and in front of the ONH. Aspiration levels were 300–600 mmHg for both vitrectors. Cut rates for the GV were 1500–5000 CPM and US power for the HV was 10%-50%. At the conclusion of the experiment, the swine were euthanized with pentobarbitone (150 mg/kg) by intra-cardiac puncture.

### Histopathological assessment

Following vitrectomy of porcine eyes, the core of the vitreous cavity was filled by 2.5% glutaraldehyde/10% formalin for 24 h, followed by immersion fixation in 10% formalin until dissection. Eyes were marked to ensure correct orientation of each eye for dissection. Samples from porcine cadaver eyes and porcine enucleated eyes which did not undergo any procedure were used as negative controls. Each eye was photographed upon dissection (MacroPATH, Milestone Srl, Italy). The eyes were horizontally sectioned at the superior edge of the cornea and any macroscopic intraocular changes noted. The eye was then horizontally sectioned through the inferior edge of the cornea. The slice between the two horizontal cuts was processed in a tissue processor (ExcelsiorAS, Thermo Scientific, Waltham, MA, USA). In short, processing consisted of dehydration in graded alcohols, delipidization in xylene and embedding in RalWax (RA Lamb, Eastbourne, UK). Subsequently serial sections were cut at 4 μ until the level of the optic disc and stained with hematoxylin-eosin and periodic acid-Schiff and Masson trichrome stains.

**Fig 9:**
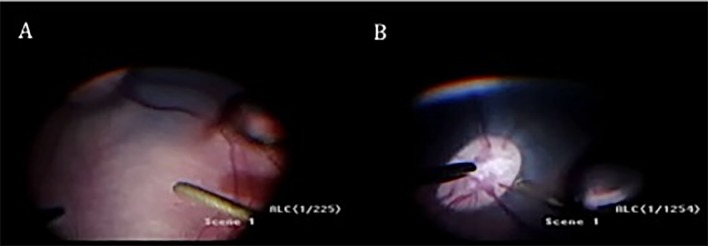
Non-recovery anaesthesia live animal experiments. (A) Shows the hypersonic vitrector over the macula and (B) Shows the hypersonic vitrector (HV) over ONH. Guillotine and hypersonic cutters were operated 3–5 mm from the retina over the macular area and the optic nerve head (ONH) for 5 minutes each (nasal area served as control).

**Fig 10:**
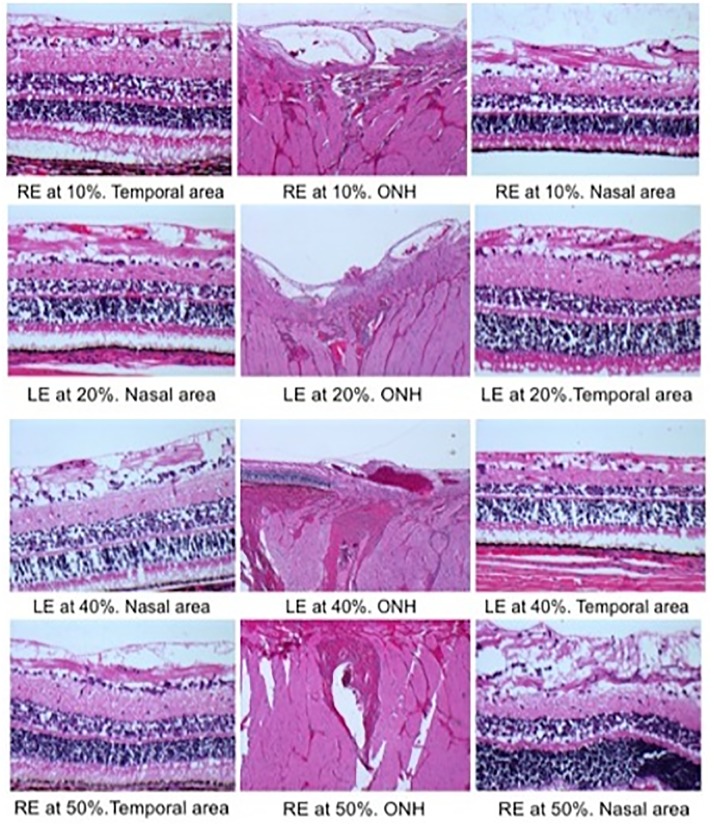
Hematoxylin-eosin stained horizontal sections in non-recovery anaesthesia live animal experiments. Hematoxylin-eosin stained horizontal sections through the optic nerve head and the areas nasal and temporal to the nerve head after pars plana vitrectomy using a hypersonic vitrector at 10%, 20%, 40%, or 50% ultrasound power. Vacuolization can be seen at all settings, as well as fragmentation of the nerve fibers and ganglion cell layers. The inner limiting membrane showed separation without disruption. (RE = right eye; ONH = optic nerve head; LE = left eye).

In addition, as all core vitrectomies were done holding the vitrector (HV or GV) 3–5 mm in front of the macula and the ONH (Fig 9A and 9B). In all of the samples, the nasal retinal areas close to the ONH also were used as a control of the macular areas in each experiment (Fig 10).

All slides were evaluated in a masked fashion for controls, vitrector type and settings by two pathologists (RB, LI) separately using a microscope (Olympus BX51, Olympus Optical Co, Tokyo, Japan). The pathologists looked for changes on the lens, vitreoretinal interface, retinal layers, choroid and ONH. Such changes could represent, but were not exclusive of findings such as intra- or extracellular edema, vascular congestion, hemorrhage and tissue disruption. Relevant microscopic features were photographed using a camera (Olympus U-CMAD3, Tokyo, Japan) and CellD imaging software (Olympus Soft Imaging Solutions GMBH, Münster, Germany).

### Electron microscopy

Human vitreous specimens were applied to freshly glow-discharged 400 mesh carbon coated copper grids, then briefly washed with distilled water, stained with 1% uranyl acetate for 30 second and blotted with filter paper. After drying, samples were observed with a Tecnai 12 Biotwin microscope (FEI, Hillsboro, OR, USA) at 100kV acceleration. Images were taken with a Orius SC1000 CCD camera (Gatan, Pleasanton, CA, USA) (Figs 7A–7F and 8A–8F).

## Results

### Porcine cadaveric eyes experiments

There were no macroscopic retinal or ONH defects associated with the use of the HV or GV. There were no differences in the microscopic retinal and ONH defects found after the use of either vitrector (Figs 3 and 4). All vitrectomized retinal specimens showed some degree of vacuolization and fragmentation at the nerve fiber layer (NFL) (Fig 3B–3F). Samples from both vitrector groups showed separation of the ganglion cell layer (GCL) and inner limiting membrane layer (ILM) (Fig 3B–3F). There were no differences between the two vitrector groups in the optic nerve head (ONH) morphological analysis (Fig 4A–4F). There was no clear correlation between US power and retinal damage

Direct touch of crystalline lenses/posterior capsule with the HV tip showed small disruptions of the posterior capsule (Fig 5B and 5D). Greater disruptions of the posterior capsule were found in samples contacted by the tip of the GV (Fig 5A and 5C).

### Human cadaver eye experiments

Hematoxylin and eosin stained specimens showed human cadaveric retinal defects (ILM fragmentation and separation and some degree of vacuolization in inner retinal layers) after GV (Fig 6A and 6B) and HV PPVs (Fig 6C and 6F). In some samples, differential interference contrast microscopy was used to highlight the ILM (Fig 6D and 6E).

The collagen that had passed through the two vitrectors was examined by electron microscopy (Figs 7A–7F and 8A–8F). Unfragmented vitreous collagen fibrils are very long and ends are rarely observed. After passing through both vitrectomy devices short lengths of collagen were observed demonstrating fragmentation. The fragments were generally shorter from the HV than the GV vitrector implying that the vitreous gel had been more efficiently disrupted.

### Live porcine eye experiments

Retinal specimens showed some degree of vacuolization at inner layers, NFL and GCL, with both vitrectors. Some cases showed ILM separation without disruption (Fig 10). However, there were no differences between the “non-vitrectomized”, nasal (control) and vitrectomized, temporal (HV) areas of the ONH or in the ONH analysis of the GV and HV.

We saw no clear correlation between the structural changes found in the inner retinal layers and the US power settings used for the hypersonic vitrectomy surgeries. Similar retinal changes were found in specimens with vitrectomy at 10% or 50% US power (Fig 10).

## Discussion

An ultrasonic surgical hand piece is primarily an acoustic assembly that includes four basic elements. These are a generator or power supply, an ultrasonic motor (transducer), a mechanical wave amplifier (referred to as an acoustic horn) and a sonotrode (or probe) [19]. There are two principal forms of vibratory energy, which have found application in medicine and surgery [20]. These are low-power high-frequency (LPHF) and high-power low-frequency (HPLF) US. Ultrasonic diagnostic imaging devices and ultrasonic physical therapy use LPHF vibratory energy (1–20 MHz), whereas, ultrasonic surgery, deploys relatively high-power (10–300W/cm2) low-frequency (20–60 kHz) vibratory energy [19–20].

Our study introduces a new technology for retinal surgery that is uniquely versatile in its capability. The HV has an ultrasonically driven handpiece with a closed end needle and a small port located on the side at the end of the needle. The HVs has only a single needle instead of two needles, this means that the port is continuously open, permitting smaller sizes on the ports and larger inner lumen diameters, therefore, providing lower flow resistance and lower infusion pressures. The HV needle is about 33 mm long, similar to the needle length of 23 gauge GV devices (Fig 1). The port diameter of the needle tested for this study was 0.007 inches and the wall thickness was 0.003inches.

The HV handpiece, contrary to the others ultrasonic surgery devices, runs with a low power harmonics US, operating at a fixed US frequency around 28.5 kHz, and a peak-to-peak amplitude between 10 μm and 50 μm.

HPLF US has wide ranging clinical applications in surgical and medical instruments for biological tissue cutting, ablation or fragmentation, and removal. However, despite its widespread clinical application and common device operating characteristics, there is an incomplete understanding of the mechanism of tissue failure, removal and damage [19].

The tissue ablation and damage mechanism is poorly understood and mechanisms for damage minimization have not been clearly defined. Moreover, our understanding of US tissue damage is predominantly subjective and based on clinical observation alone [19–24]. In addition, the literature reporting the mechanism of interaction is limited and frequently conflicting [19, 21–23]. This incompleteness in understanding underpins the reticence of some clinicians to embrace this technology, as the idea of US vitrectomy was first introduced by Girard in 1975 [25].

The potential US damage mechanisms occurring in tissues include alteration in global biomechanical properties, histomorphological changes, protein denaturation and tissue necrosis [15–17, 19, 26].

In our study, compared to control eyes, porcine and human cadaveric eyes that had undergone PPV with GV or HV had retinal lesions primarily at the inner retinal layers, with some degree of vacuolization and fragmentation in the NFL and GCL, as well as ILM separation (Figs 3 and 6). These observed retinal changes (vacuolization and fragmentation) could be related to normal postmortem changes [27] or to excessive amount of suction during vitrectomy procedures [28] as the histological analysis (qualitative assessment) under LM in the HV samples did not reveal extensive cellular injuries, thermal damage in inner and outer retinal layers or extensive tissue coagulation and trans-mural vessel necrosis. Neither did we find differences between the open-sky or closed vitrectomy samples (Figs 3, 4 and 6).

No morphological differences in retinal histopathology were observed between HVs and GVs after PPV *in vivo* (Fig 10). Macro- and microscopic histopathological findings were nonspecific, showing some degree of vacuolization at inner retinal layers especially in the HV samples. This vacuolization could be related with the “cavitation” phenomena, which can occur at intra-cellular or extra-cellular level, causing cell fragmentation and cell destruction or in the surrounding fluid, causing inefficient coupling with energy dissipated and no cellular fragmentation [19]. Subjectively, the qualitative histological assessment did not reveal extensive cell fragmentation or destruction in the HV samples. Moreover, all of these retinal changes appeared at the inner retinal layers, without finding any deleterious effect on the RPE layer such as that seen in previous studies [15–17, 26] (Fig 10). These retinal changes in our study, may have been minimized by the small diameter of the tip and port of the HV (port diameter (0.007 inches)/Wall thickness (0.003inches) and the low US power (less than 5% of the US energy used in traditional lens fragmentation) used in the current study (Fig 1).

To our best knowledge, the structural changes in the vitreous after vitrectomy have never been reported at the TEM level. The vitreous collagen fibrils are normally very long and thin and it is these that are responsible for maintaining the gel state of the vitreous [29–30]. According to our results, more fragmentation of the vitreous collagen fibers and less aggregation of residual collagen network around these fragments were shown in the HV samples (Figs 7 and 8). Such increased operational efficacy could result in a change in the rheological properties of the chopped vitreous (decreasing both its elasticity and viscosity), improving its flow inside the vitrectomy system [7, 31].

In addition, greater disruptions of the posterior capsule were found in samples contacted by the tip of the GV (Fig 5A and 5C). The response of biological tissue to ultrasound can be quite variable and depends on the acoustic and biological properties as well as on location and function of exposed tissue [32].

It has been established that absorption coefficient increases as a function of protein content, with collagen having particularly high specific absorption [33]. In this regard, it should be pointed out that total amount of protein and in particular collagen in vitreous is less than in the lens [34–35], in which one of the major capsular proteins is collagen [35]. Biological tissues with higher collagen content better withstand the vibratory insult form ultrasonic energy and do not fragment, whereas "weaker" tissues will. In comparison to harder tissues, soft tissues are highly compliant and large amplitude and high-frequency vibrations can be used to fragment soft tissue easily [19, 21, 36]. Therefore, the total amount of collagen, the tip and port configuration of the HV and the reduced level of US power used may have influenced in the results obtained in the lens experiments (Fig 5A and 5C).

There are limitations to this study. Although cadaveric eyes were obtained within 12–24 h of slaughter, the observed retinal changes could be related to normal postmortem changes [27] or to the harvesting, packing, shipping and handling of the eyes. Also, a disciform lesion secondary to neovascular age macular degeneration (nAMD) (Fig 6F) was found in one of the human samples. Thus, the samples may not have been representative of the normal population undergoing vitrectomy.

All the swine were sacrificed immediately after the surgical procedures, so at present we can not know if there are long-term effects associated with use of intravitreal US, neither electroretinography or visual evoked potentials (VEPs) tests were performed to assess the functionality of retina cells and optic nerve fibers after HV vitrectomy.

In this first report, we have focused our attention on the histomorphological changes (qualitative assessment), but not in the global biomechanical properties, protein denaturation or molecular changes. Further quantitative evaluations of US tissue effects and technological improvements on the HV are currently ongoing which should ultimately contribute to increase the efficacy of this prototype.

In addition, regarding to TEM experiments, due to the scarcity of human samples, we have compared our results in vitreous gel with those obtained in previous studies in non-vitrectomized human vitreous samples [29–30]. In addition, the collagen fibers were visualized by using standard contrasting methods preventing the correct assessment and visualization of molecular links as proteoglycans in the collagen network after vitrectomy.

In summary, to our knowledge the structural changes in the vitreous after PPV have never before been evaluated at electron microscopic level. The fragmentation of the collagen vitreous with the HV appeared to be more effective, compared to the GV. Although these first results might lead us to consider the HV as a new promising technology for PPV, further studies are required to analyse thoroughly and quantitatively its safety and efficacy in order to avoid serious and preventable adverse effects.
